# Longitudinal cerebrospinal fluid assessment in a patient with tuberculous meningitis—A case report

**DOI:** 10.1002/jcla.23286

**Published:** 2020-03-11

**Authors:** Yuxin Chen, Xiaojin Liu, Xun Zhang, Zhihua Zhang, Xueqin Zhou, Yuqing Wang, Shucai Wu, Liheng Zheng

**Affiliations:** ^1^ Department of Laboratory Medicine Nanjing Drum Tower Hospital Nanjing University Medical School Nanjing China; ^2^ Department of Infectious Disease Fifth Hospital of Shijiazhuang Shijiazhuang China; ^3^ Department of Laboratory Medicine Chest Hospital of Hebei Province Shijiazhuang China; ^4^ Department of Respiratory Medicine No.4 people’s hospital of Qinghai Province Xining China; ^5^ Department of Tuberculosis Chest Hospital of Hebei Province Shijiazhuang China

**Keywords:** cerebrospinal fluid, cytological analyses, tuberculous meningitis

## Abstract

**Background:**

Dynamic assessment of cerebrospinal fluid (CSF) is essential for diagnosis, treatment, and prognosis of tuberculous meningitis, one of the most severe forms of central nervous system (CNS) infection.

**Case presentation:**

A 45‐year‐old man sought care as he developed confusion, clonic convulsion, and coma. Longitudinal, comprehensive analyses of cytological, biochemical, and microbial changes in CSF specimen were assessed for this patient. On day 1 of hospitalization, modified Ziehl‐Neelsen staining of CSF identified positive acid‐fast bacilli, cytological analysis revealed neutrophilic‐predominant pleocytosis (neutrophils 77%), and adenosine deaminase (ADA) was substantially elevated. Therefore, tuberculous meningitis was diagnosed and first‐line standard anti‐tuberculosis treatment was initiated. Interestingly, after 7‐day treatment, the patient was greatly improved, and CSF disclosed a dominant percentage of lymphocytes (82%) as well as macrophages engulfing *Mycobacterium tuberculosis*. Later, the dose of dexamethasone was reduced, large number of neutrophils (57%) was present and protein level was immediately elevated in CSF specimen, indicating a possible relapse of tuberculous meningitis. Since the clinical condition of the patient was not worsening, the patient was stick to reduced dose of dexamethasone and standard anti‐tuberculosis agents. He was discharged from the hospital on day 34, with 1‐year continuation standard anti‐tuberculosis therapy, and was clinically resolved from tuberculous meningitis.

**Conclusion:**

Detailed analyses of cellular composition, biochemical results, and microbial tests of CSF specimen provide the physician direct evidence of the immune surveillance status during tuberculous meningitis, which facilitates early diagnosis, optimal treatment, and improved prognosis.

**Abbreviations**
AFB: acid‐fast bacilli
CNS: central nervous system
CSF: cerebrospinal fluid
MRI: magnetic resonance imaging
MTB/RIF: *Mycobacterium tuberculosis*/rifampicin
WBC: white blood cell count

## 1 BACKGROUND

Tuberculous meningitis is the most severe form of tuberculosis, leading to devastating clinical outcome. Timely diagnosis and immediate anti‐tuberculosis treatment are vital to reduce morbidity and mortality. For central nervous system (CNS) infection, dynamic assessment of cerebrospinal fluid (CSF) is essential for diagnosis, treatment, and prognosis of tuberculous meningitis. In this case, we monitored cytological, biochemical, and microbial changes of CSF from a patient with tuberculous meningitis, which highlight the clinical significance of longitudinal CSF analysis during tuberculous meningitis.

## 2 CASE PRESENTATION

**Figure 1:**
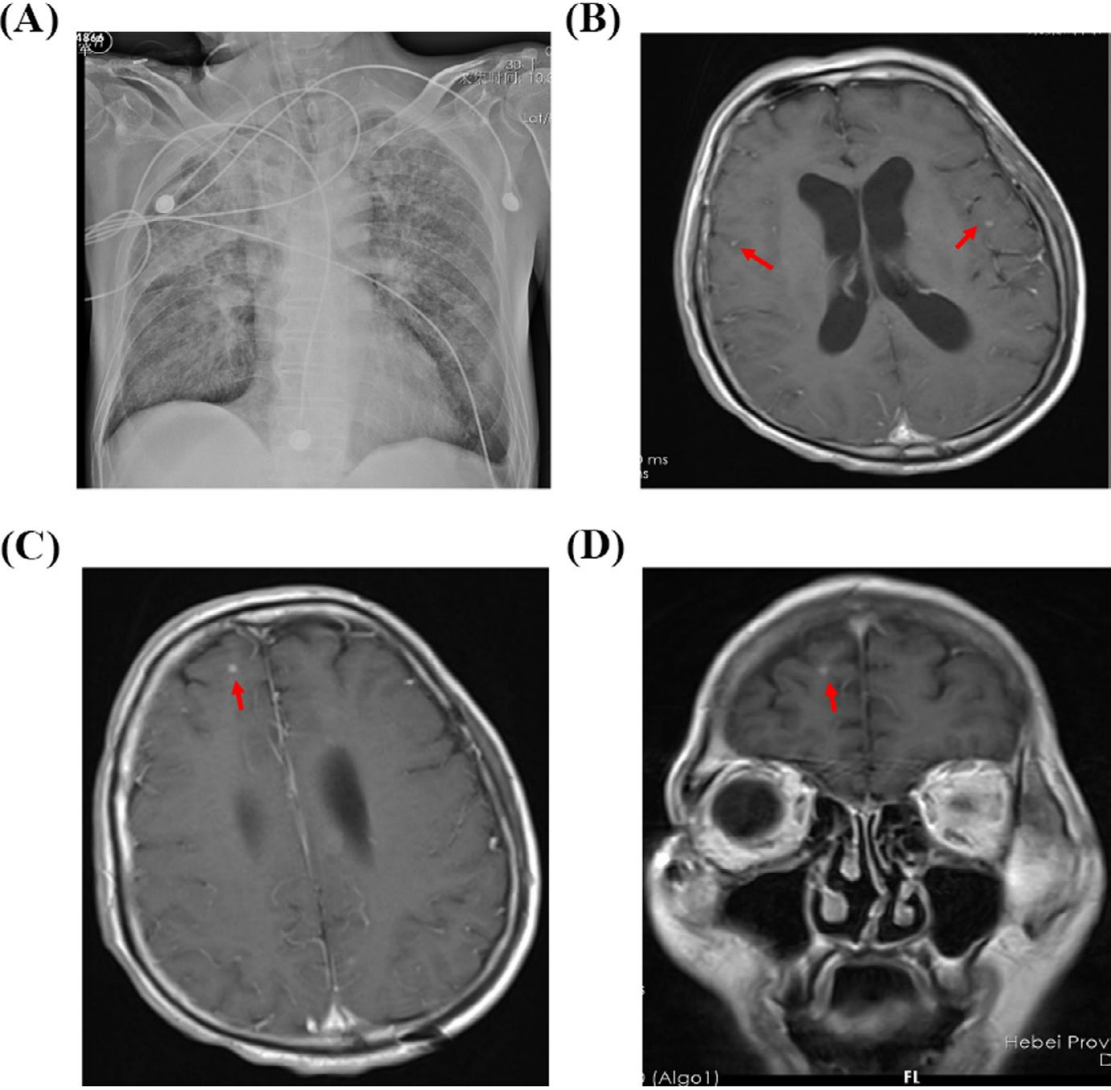
Chest X‐ray and cranial magnetic resonance imaging (MRI) of the patient with tuberculous meningitis. A, Chest X‐ray showed multiple patches and nodules with observable cavity in his lungs. B‐D, T1‐weighted image showed enhanced nodules in gray matter and white matter border at the right frontal region, as well as lateral meningeal enhancement at the left temporal lobe

**Figure 2:**
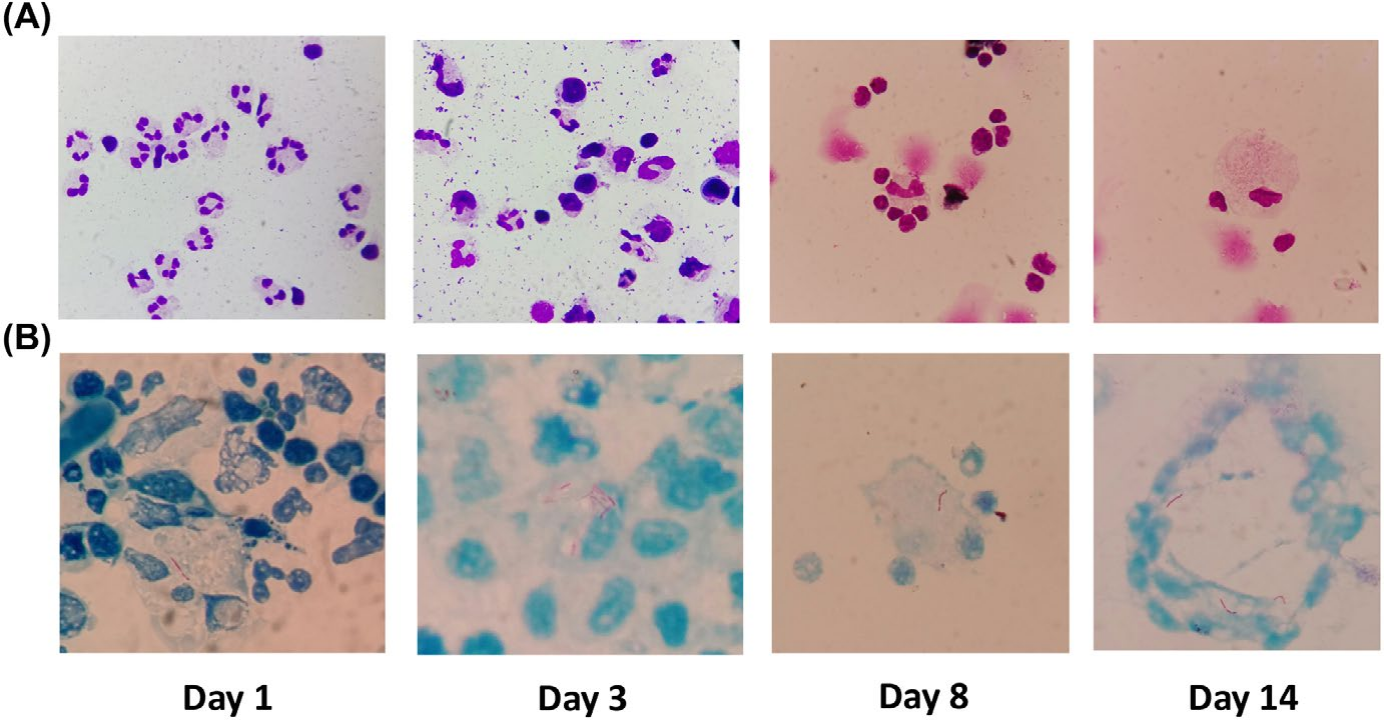
Dynamic cytology of cerebrospinal fluid assessment at day 1, day 3, day 8, and day 14

A 45‐year‐old man with a history of alcohol‐use disorder and altered mental status was hospitalized due to confusion and clonic convulsion. Three days later, this patient was transferred to our hospital. On examination, his body temperature was 38.4°C. The patient fell into coma, and his pupils were equal, round, and reactive to light. Pulmonary moist rales, neck stiffness, and limited limb strength were presented. Chest X‐ray showed multiple patches and nodules with observable cavity in his lungs (Figure 1A). The enlargement of the bilateral ventricle was observed in cranial magnetic resonance imaging (MRI). T1‐weighted image showed enhanced nodules in gray matter and white matter border at the right frontal region, as well as lateral meningeal enhancement at the left temporal lobe (Figure 1B‐D). A lumbar puncture was successfully performed and revealed an opening pressure of 35 cm H_2_O. Analysis of CSF showed a neutrocytic pleocytosis (white blood cell count [WBC] 236 × 10^6^/L, 77% neutrophils) (Figure 2A). Further, reduced glucose of 1.26 mmol/L, declined chlorine of 108 mmol/L, and remarkable increased protein of 2.17 g/L were observed in CSF (Table 1). Biochemical analyses also showed substantial increased ADA of 58.7 U/L. Using modified Ziehl‐Neelsen staining,^1^ few acid‐fast bacilli (AFB) were detected in his CSF (Figure 2B), while Gram staining and India ink preparations showed no microorganisms. All these laboratory results lead to clinical diagnosis of tuberculous meningitis. He was immediately administered with rifampin (600 mg/d), isoniazid (600 mg/d), pyrazinamide (1500 mg/d), ethambutol (750 mg/d), and dexamethasone (10 mg/d).

**Table 1:** Longitudinal biochemical, cytological, and microbiological results of cerebrospinal fluid from the patient

Variable	Day 1	Day 3	Day 5	Day 8	Day 11	Day 13	Day 22	Day 27	Day 34	Day 45
Opening pressure (cm of water)	35	40	19	15	9	8.5	4	5	NA	NA
Color	Light yellow	Light yellow	Yellow	Yellow	Light yellow	Light yellow	Light yellow	Light yellow	Light yellow	Light yellow
Turbidity	Mild turbid	Mild turbid	Clear	Turbid	Clear	Clear	Clear	Clear	Clear	Clear
White blood cells (10^6^/L)	236	226	151	643	203	238	141	152	682	322
Neutrophils (0%‐6%)	77	27	14	6	4	1	4	10	57	14
Lymphocytes (40%‐80%)	20	67	78	82	82	95	90	88	42	84
Monocyte (15%‐45%)	3	6	8	12	14	4	6	2	1	2
ADA (0‐5 U/L)	58.7	73	40	21	13	3.6	3	5	8	6.9
Protein (<0.45 g/L)	2.17	2.02	1.92	1.73	1.57	1	1.74	1.56	1.9	1.8
Glucose (2.2‐3.9 mmol/L)	1.26	1.23	1.89	1.71	2.2	2.13	1.16	1.77	2.3	1.9
Chloride (120‐140 mmol/L)	108	106	113	108	106	113	0.86	116	111	110
Microbiological testing	+	+	+	+	+	+	+	−	−	−

On day 3 of admission, clinical assessment of the patient showed unconsciousness, tachycardic (plus 130 beats/min), with a respiratory rate of 35 breaths/min. The patient had an excessive sputum but with difficulty to cough up. He needed a 10 L/min oxygen via a face mask, resulting in an arterial PaO_2_ of 6.8 kPa. The patient still presented substantial hypoxemia (SpO_2_ 78%), necessitating oral incubation and mechanical ventilation. A repeat lumbar puncture showed an increased opening pressure (40 cm H_2_O), pleocytosis (WBC 226 × 10^6^/L, 27% neutrophils) (Figure 2A). During the following 5 days, the lumbar puncture still showed a high opening pressure, dynamic fluctuated WBC with gradually declining neutrophils to 5.8% (Table 1). However, lumbar CSF tested positive for *Mycobacterium tuberculosis* by both modified acid‐fast staining and Xpert *Mycobacterium tuberculosis*/rifampicin (MTB/RIF) assay. On day 8, the patient's mental status progressively improved. He was able to blink, and no neck stiffness was present. CSF analysis also revealed almost disappearance of neutrophils. However, AFB staining was still positive (Figure 2B).

Day 9, the patient became alert and oriented. CSF revealed progressively declined ADA and protein level (Table 1). Oral incubation and mechanical ventilation were removed. The patient could open his eye on his own on day 11. Despite his speech ability was improved, his speech output was irrelevant. He was able to move his limbs spontaneously and against gravity. Lumbar CSF on day 14 showed a progressively declining pleocytosis with smaller percentage of neutrophils (4%) and increased percentage of macrophages (14%) engulfing *Mycobacterium tuberculosis*. Elevated ADA level and AFB were detected again (Figure 2).

On day 17, the patient clinically improved and speech ability was fully recovered. For the first time, AFB were not detected. Cytological examination of CSF disclosed a lymphocytes‐predominant pleocytosis. Given such results, the physician lowered the dose of dexamethasone to 6 mg. After 3 days of reduced dexamethasone treatment, neutrophils‐dominating white blood cells (57% neutrophils) were found and protein was dramatically increased, indicating a relapse of tuberculous meningitis. However, clinical assessment of the patients was not worsening. Therefore, the patient was stick to 6 mg of dexamethasone and standard first‐line anti‐tuberculosis drugs. He was discharged from the hospital on day 34, with 1‐year continuation standard anti‐tuberculosis therapy, and was clinically resolved from tuberculous meningitis.

## 3 DISCUSSION AND CONCLUSIONS

Meningitis is the most severe form of tuberculosis, result in mortality or neurological disability in 50% of patients.^2^, ^3^ Nevertheless, *Mycobacterium tuberculosis* is a cytozoic pathogen and its numbers are very few in cerebrospinal fluid.^4^ Therefore, detecting *M tuberculosis* in CSF from tuberculous meningitis patients is still a challenge for clinicians.^5^ As the cornerstone of TBM diagnosis, the sensitivity of Ziehl‐Neelsen stain of CSF is low. In our case, here, we are using a modified Ziehl‐Neelsen stain with high sensitivity and accuracy for both intracellular and extracellular AFB CSF microbiological analysis.^1^, ^6^ On day 1 of hospital admission, we were able to find 1 or 2 AFB in visual fields, an important microbiological evidence that ultimately leads to the diagnosis of tuberculous meningitis. Due to this prompt diagnosis, the patient received first‐line anti‐tuberculosis treatment immediately and fully resolved from tuberculous meningitis later.

Longitudinal evaluation of CSF specimen is critical to monitor disease progression of tuberculous meningitis. A compressive CSF assessment includes biochemical, cytological, and microbiological results. We closely and comprehensively analyzed CSF specimen from day 1 until day 45 since hospitalization of the patient. First, in our case, the microbiological test revealed by modified Ziehl‐Neelsen stain results in prompt diagnosis and treatment. AFB was not detected cleared until day 27 post‐treatment, along with the alleviation of cytological and biochemical laboratory parameters. Furthermore, since CSF is an immunologically active body fluid, cytological evaluation of CSF specimen provides important information over the disease progression. On CSF examination at day 1, we found dominant presence of neutrophil granulocytes, indicating the patient underwent acute phase of tuberculous meningitis.^7^ After 7‐day treatment, the patient was greatly improved, and CSF disclosed a dominant percentage of lymphocytes (82%) as well as macrophages engulfing *Mycobacterium tuberculosis*, suggesting the activation of immune surveillance in CNS.^8^ Interestingly, when the patient was treated with reduced dose of dexamethasone, neutrophil granulocytes were dramatically increased to 57% in CSF within 2 days. While the patient clinically improved on day 45, the neutrophils were declined to 14%.

The role of neutrophils remains controversial in pathogenesis of tuberculous meningitis.^9^, ^10^ It is considered that neutrophils are typically the first responder in host defense at the site of tuberculosis. A previous case report also noted that predominately neutrocytic pleocytosis present in CSF strongly suggested tuberculous meningitis.^11^ Further, a cohort study recently identified that mortality was associated with higher CSF neutrophil counts.^12^, ^13^ Indeed, neutrophils accumulate in response to uncontrolled inflammation and might cause tissue damage via release of neutrophil‐associated mediators that exacerbate clinical deterioration.^14^ Therefore, measurement of the neutrophils in CSF specimen is crucial to evaluate the disease progression of tuberculous meningitis.

Traditional laboratory tests including ADA of CSF are also helpful for diagnosis and prognosis. As an enzyme wildly presented in tissues and body fluid, ADA has been routinely used in *Mycobacterium tuberculosis*. Despite ADA test has high sensitivity and specificity, the diagnostic value of CSF ADA remains to be explored. It is necessary to interpret the ADA result with the combination of clinical symptoms and laboratory results.^15^ In our case, with the clinical improvement of tuberculous meningitis, ADA level was gradually declined along with reduced protein, whereas the lymphocytes were dominated in the CSF.

To summarize, detailed analyses of cellular composition, biochemical results, and microbial tests of CSF specimen provide the physician direct evidence of the immune surveillance status during tuberculous meningitis, which facilitates early diagnosis, optimal treatment, and improved prognosis.

## ETHICS APPROVAL AND CONSENT TO PARTICIPATE

Not applicable.

## CONSENT FOR PUBLICATION

Written informed consent was obtained from the patient for publication of this Case report and any accompanying images.

## DATA AVAIALBILTY STATEMENT

All relevant data and materials are included in the manuscript.
